# The radiological impact of phosphogypsum stockpile in Wiślinka (northern Poland) on the Martwa Wisła river water

**DOI:** 10.1007/s10967-015-4191-5

**Published:** 2015-06-25

**Authors:** Grzegorz Olszewski, Alicja Boryło, Bogdan Skwarzec

**Affiliations:** Laboratory of Analytical and Environmental Radiochemistry, Department of Environmental Chemistry and Radiochemistry, Faculty of Chemistry, University of Gdańsk, 80-308 Gdańsk, Poland

**Keywords:** Polonium, Uranium, Radiolead, Phosphogypsum, River water

## Abstract

The aim of this work was to determine the concentrations of uranium (^234^U, ^235^U, ^238^U), polonium (^210^Po) and lead (^210^Pb) radioisotopes in water samples and to explore the impact of the phosphogypsum stack on the Martwa Wisła waters. The ^238^U, ^210^Po and ^210^Pb concentrations in analyzed water samples reached maximum values of 11.7 ± 0.3, 2.0 ± 0.1 and 3.2 ± 0.1 mBq L^−1^ and activity ratios were maximally 1.18 ± 0.01 for ^234^U/^238^U, 0.041 ± 0.018 for ^235^U/^238^U and 0.69 ± 0.10 for ^210^Po/^210^Pb. The obtained results suggest that this impact is rather insignificant and does not affect significantly the Martwa Wisła river.

## Introduction

The phosphogypsum waste heap in Wiślinka is one of the most significant components of the environment of the Vistula river delta in northern Poland. Its location between the Martwa Wisła river and farm fields could have negative environmental impact on approximate areas, including Gdańsk agglomeration. The phosphogypsum stockpile in Wiślinka is the result of phosphorite fertilizers deposition produced by Phosphoric Fertilizers Industry in Gdańsk. Phosphoric acid, the material for the production of phosphate fertilizers, is obtained in a wet process by reaction of the phosphatic rocks with sulphuric acid. Phosphogypsum (CaSO_4_·2H_2_O) is a by-product of this reaction [1]. Phosphogypsum stockpile in Wiślinka contains about 16 million ton of phosphogypsum [2]. The essence of radiotoxicity of the phosphogypsum is both gamma radioactivity and high content of natural alpha radioactive elements (^234^U, ^235^U, ^238^U, ^226^Ra, ^210^Po) and also beta emitter (^210^Pb), which could be leached by rain and bioaccumulated in plants and animals as well as in humans. In the process of phosphoric acid production about 80 % of uranium is associated with the phosphoric acid fraction, while about 90 % of the ^210^Po and ^210^Pb is bound to the phosphogypsum fraction [1, 3]. Radionuclides might be leached by wet precipitation and transported through groundwaters to plants where they are accumulated [2, 4–8].

Natural uranium consists of three alpha radioactive isotopes: 99.2745 % of ^238^U, 0.7200 % of ^235^U, and 0.0054 % of ^234^U [9]. The occurrence of uranium in environment can be a result of human activities like nuclear industry, combustion of fossil fuels, production and use of phosphorous fertilizers, use of depleted uranium for military purposes [10–12]. The ^234^U and ^238^U radionuclides are not in the radioactive state of equilibrium in water environment. Similar disequilibrium is noticed between ^235^U and ^238^U and the values of this ratio is connected with the use of nuclear weapons, pollution associated with nuclear power and using missiles with depleted uranium [13]. In groundwaters the average values of the activity ratio between ^234^U and ^238^U are in the range from 0.51 to 9.02, while in salt water from 1.11 to 5.14, in river water from 1.00 to 2.14, in river suspension from 0.80 to 1.00, in oceanic water 1.14 and in Baltic water 1.17 [14–16]. Natural value of the ^235^U/^238^U activity ratio is close to 0.046 [13].

The natural radionuclides ^210^Po and ^210^Pb are daughters of ^238^U decay series with half-lives of 138.38 days and 22.3 years, respectively. These natural radionuclides are found in varying concentrations in soil, sand, sediment and natural water and constitute an important component of the natural background radiation. ^210^Po and ^210^Pb radionuclides are well known to significantly contribute to the radiation dose of the population as they are easily dissolved in water [17, 18]. The main source of ^210^Po and ^210^Pb in the atmosphere is ^222^Rn emanation from the ground. ^210^Po and ^210^Pb return to the earth as dry fallout or are washed out in rain. Important anthropogenic sources of these radionuclides are burning of fossil fuels and tetraethyl lead in petrol, dust storms, refineries, superphosphate fertilizers, the sintering of ores in steelworks, the burning of coal in coal-powered power stations [7]. The possible different chemical behavior of both ^210^Po and ^210^Pb in the water column is characterized by a stronger affinity of ^210^Po for particles than its precursor, ^210^Pb [19]. The usual atmospheric input by rain has ^210^Po/^210^Pb ratio of 0.1–0.2 [20]. Ratio higher than 1.0 must be affiliated with bio-geochemical processes that can control the distribution of the two radionuclides [20]. The purpose of this study was to examine the actual impact of the possible radionuclides containing leachates from phosphogypsum stack and to assess background levels of natural radionuclides to monitor the changes of their concentration in future surveys.

## Experimental

**Fig. 1:**
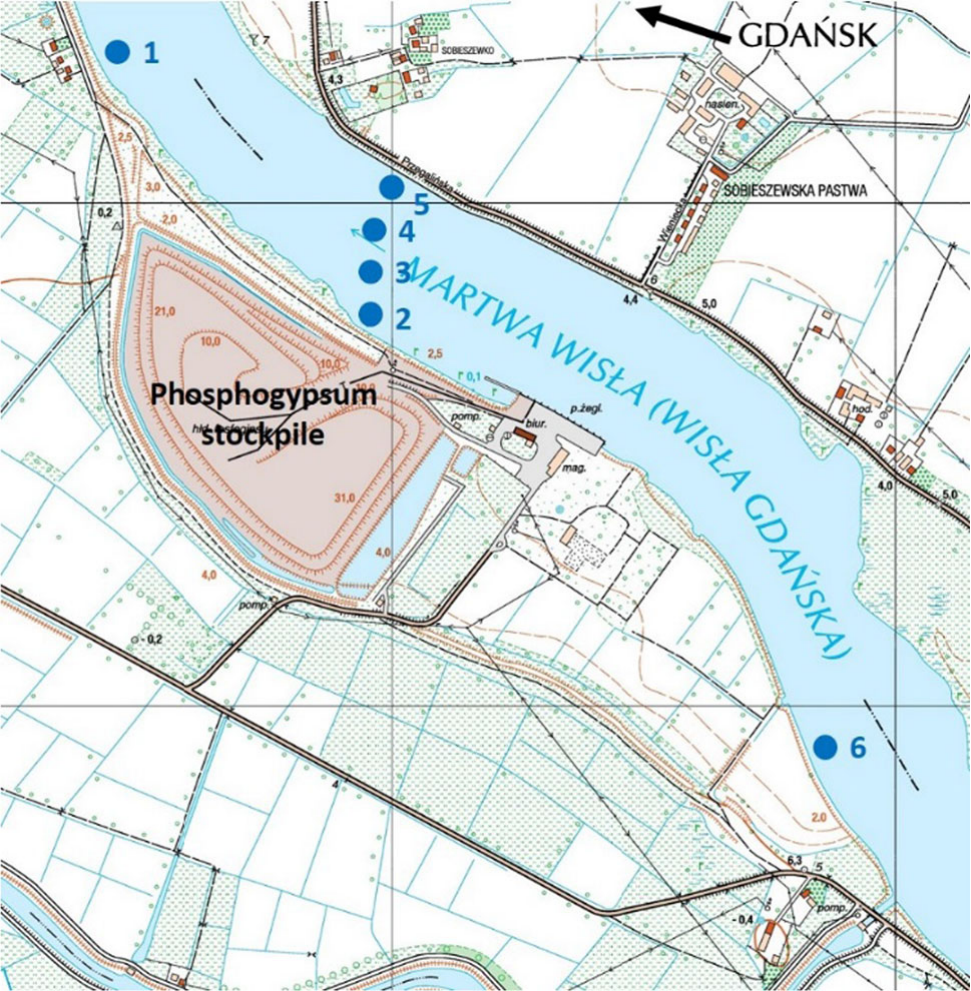
Water samples collection sites

Surface river water samples (10 L) were collected from the Martwa Wisła river in October 2013. Sample location sites are presented on Fig. 1. Samples were collected along the shore of the river and in its gradient. Waters were collected in polyethylene bottles. Before analysis to each sample yield tracers (^209^Po and ^232^U) were added and the water was filtered through Whatman 0.45 µm filters. All the nuclides were coprecipitated with MnO_2_ that was later dissolved in HCl/H_2_O_2_ solution. Polonium was electrodeposited on silver discs and uranium after separation on Dowex anion-exchange resins, was electrolyzed on stainless steel discs according to the procedure by Skwarzec and Boryło [21, 22]. ^210^Pb was analyzed indirectly through its daughter ^210^Po after 6 months storage. After this time polonium was again electrodeposited on silver disc and ^210^Pb activity was calculated through ^210^Po activity [21].

The activities of ^210^Po, ^234^U, ^238^U and ^235^U were measured using an alpha spectrometer (Alpha Analyst S470) equipped in a surface barrier PIPS detector with an active surface of 300–450 mm^2^ placed in a vacuum chamber connected to a 1024 multichannel analyzer (Canberra–Packard, USA. Detector yield ranged from 0.30 to 0.40. In most of the used detectors with a surface of 300–450 mm^2^ the resolution was 17–18 keV. Minimum detectable activity (MDA) for ^210^Po and ^210^Pb were 0.03 and 0.034 mBq L^−1^, while those for ^234^U, ^235^U and ^238^U were 0.02, 0.01 and 0.02 mBq L^−1^. The accuracy and precision of the radiochemical method were estimated to be less than 7 % by participation in international intercomparison exercises and analysis of IAEA materials. The precision between subsamples was estimated to be less than 3 % for all analyzed radioisotopes. ^210^Po activities were corrected for decay between deposition on silver discs and counting on alpha spectrometer. ^210^Po and ^210^Pb were not corrected for sampling date as time between sampling and first deposition did not exceed 5 days.

## Results and discussion

### Polonium (^210^Po) and lead (^210^Pb) analysis

**Fig. 2:**
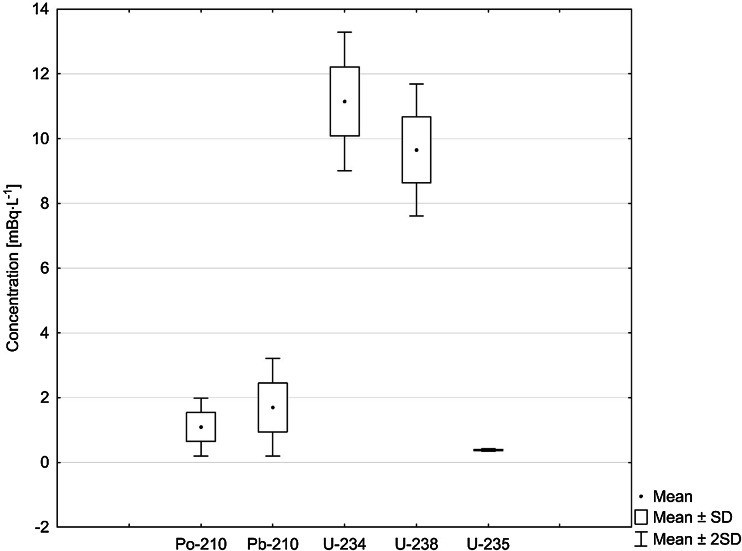
Boxplot for analyzed radioisotopes concentration in surface water samples

**Fig. 3:**
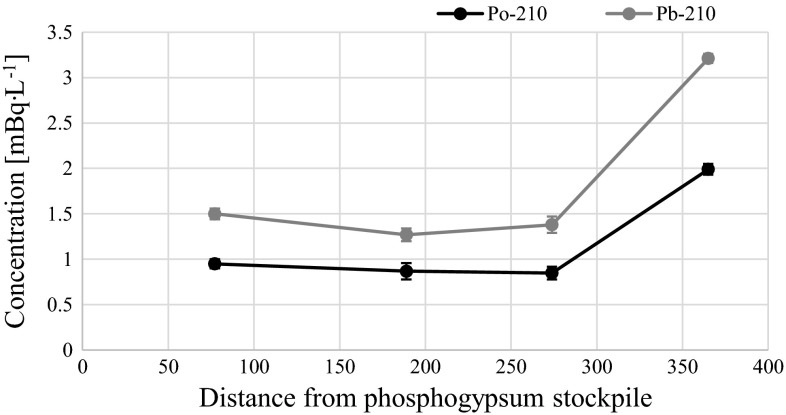
^210^Po and ^210^Pb concentrations in river gradient

**Fig. 4:**
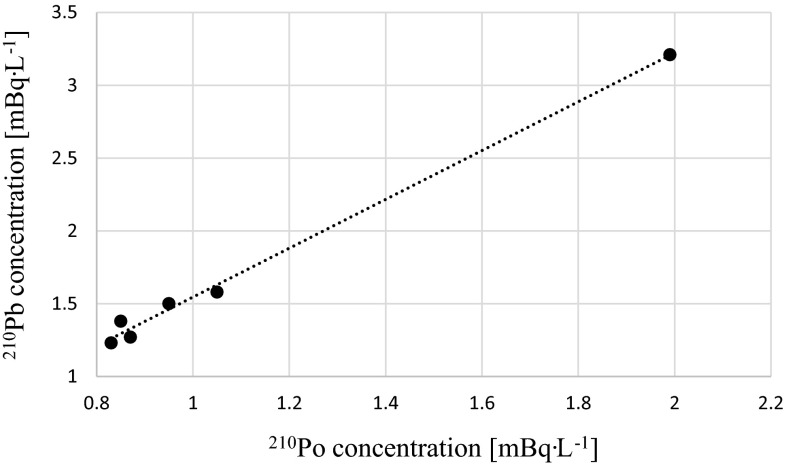
Correlation between ^210^Po and ^210^Pb concentrations in analyzed water samples (*r*_s_ = 0.94)

General overview on analyzed radioisotopes activities in sampling locations are presented on Fig. 2. The activities of ^210^Po and ^210^Pb radioisotopes in Martwa Wisła river are given in Table 1. Polonium (^210^Po) concentrations in analyzed water ranged from 0.8 ± 0.1 to 2.0 ± 0.1 mBq L^−1^ while radiolead (^210^Pb) concentration lied between 1.2 ± 0.1 and 3.2 ± 0.1 mBq L^−1^ (Table 1). Taking into account that one of the main aims of this study is to establish the impact of the phosphogypsum stack on the river waters, we cannot distinguish any polonium or radiolead enrichment close to the heap. Concentrations of ^210^Po and ^210^Pb received for the samples collected 1200 m upstream of stockpile (sample number 6), in the vicinity of the stockpile and 800 m downstream of stockpile (sample number 1) (Fig. 1; Table 1) suggest that there are no significant leachates containing these radioisotopes. The highest concentration of ^210^Po and ^210^Pb radionuclides was measured in water collected from the opposite bank of the river (Figs. 1, 3; Table 1). This fact may suggest that there is another point source of polonium and radiolead that cannot be clearly defined without further research. This might be connected with the fact that close to this bank of the river Sobieszewko and Sobieszewska Pastwa villages are located and rainfall waters can be stored and washed from here into the river through roads or pavements [8]. What is more, as we found in our previous research on ^210^Po and ^210^Pb determination in soils collected nearby stockpile, on this bank of the Martwa Wisła river sand soil is dominant. This type of soil is well known for its low retention of radionuclides due to the low organic matter content [2]. Previous survey conducted in this area in 2008 revealed higher concentrations of ^210^Po radioisotope in surface river water (from 7.08 ± 0.52 to 12.26 ± 2.2 mBq L^−1^). In other locations like stockpile’s retention reservoir or pumping station ^210^Po concentrations were 114.8 ± 5.8 and 131.4 ± 0.9 mBq L^−1^, respectively [6, 8], but you should take into attention that unfiltered water has been studied. About 60 % of ^210^Po is associated with particulate matter [20], so we can assume that ^210^Po concentration in diluted fraction of the Martwa Wisła river has decreased in time. The obtained results of polonium concentration from the Martwa Wisła water samples are similar to these from other major Polish rivers, where the activities of ^210^Po in autumn in unfiltered water were: from 1.51 ± 0.04 to 4.13 ± 0.03 mBq L^−1^ for the Vistula river; from 1.65 ± 0.03 to 8.93 ± 0.03 mBq L^−1^ for the Vistula drainage basin; from 1.25 ± 0.08 to 1.64 ± 0.08 mBq L^−1^ for the Oder river and from 1.09 ± 0.03 to 5.21 ± 0.19 mBq L^−1^ for the Oder river drainage [23]. Amounts of ^210^Po and ^210^Pb in environmental samples collected nearby phosphogypsum stacks are dependent on these radionuclides content in phosphogypsum. We found that average ^210^Po and ^210^Pb concentrations in phosphogypsum from Wiślinka stockpile are 946.7 ± 12.3 and 941.4 ± 14.9 mBq g^−1^ dry wt., respectively. Compared to other activities of dissolved ^210^Po and ^210^Pb concentrations our results are very similar. In case of Venice lagoon ecosystem where phosphogypsum stockpile impact was also examined ^210^Po and ^210^Pb concentration were 0.58 ± 0.35 and 0.64 ± 0.28 mBq L^−1^, respectively and the authors did not find any direct impact of phosphogypsum on river waters [20]. The results for the Kaveri river in India were 2.74 ± 0.53 mBq L^−1^ for ^210^Pb and 1.10 ± 0.28 mBq L^−1^ for ^210^Po [24, 25]. For the Kali, the Sharavathi and the Netravathi rivers mean concentrations of ^210^Po and ^210^Pb were 1.28 ± 0.2 and 1.37 ± 0.2 mBq L^−1^, 1.30 ± 0.2 and 1.44 ± 0.2 mBq L^−1^, 1.00 ± 0.2 and 1.22 ± 0.2 mBq L^−1^, respectively [17]. According to the authors of that paper, the presence of ^210^Po and ^210^Pb in surface waters is mainly connected with air deposition, leaching from sediments, soils, rocks and from agricultural regions, especially during heavy rains [17, 26]. Increased values of ^210^Pb concentrations were measured in the Odiel and the Tinto rivers in Huelva, Spain. Negative impact of the nearby phosphoric acid industrial complex was confirmed during three sampling campaigns. Radiolead concentrations varied from 7.4 ± 1.1 to 601 ± 43 mBq L^−1^ [27]. Borges et al. conducted ^210^Pb analysis in the filtered surface water samples from stream in city of Imbituba in Brazil. ^210^Pb concentrations ranged from 15 ± 3 to 135 ± 7 mBq L^−1^. These results are much higher than in the Martwa Wisła river. The authors explain that the highest result (135 ± 7 mBq L^−1^) is connected with effluents discharge near this sampling location [28]. The values of ^210^Po/^210^Pb activity ratios in the Martwa Wisła ranged between 0.62 ± 0.07 and 0.69 ± 0.01. These activity ratios are typical for waters because the reported values for fresh water are around 0.4–0.5 [29]. For comparison in surface water samples from the Kali, the Sharavathi and the Netravathi rivers in India the values of ^210^Po/^210^Pb activity ratios were 0.93, 0.89 and 0.85, respectively [17]. Similar value was obtained for waters from Venice lagoon (0.87 ± 0.18) [20]. Disequilibrium between these two radioisotopes exists despite the fact that both ^210^Po and ^210^Pb are particle reactive what suggests that ^210^Po is adsorbed by particles with higher efficiency. Polonium can be taken up by bacteria’s or algae. This can contribute to its faster removal from water [19, 30]. The analysis of activity disequilibrium values indicates the similar major source of ^210^Po and ^210^Pb radioisotopes in the Martwa Wisła river water. The value of ^210^Po/^210^Pb average activity ratio in phosphogypsum is 1.00 ± 0.02. Based on these results we can assume that the value of ^210^Po/^210^Pb in the Martwa Wisła river should be increased if phosphogypsum stockpile impact was substantial. It is confirmed that also some natural processes can contribute to significant change of the value of ^210^Po/^210^Pb activity ratio as possible atmospheric input by rain gives a ^210^Po/^210^Pb ratio of 0.1–0.2 [20]. The scatter plot (Fig. 4) between concentration ^210^Po and ^210^Pb radionuclides shows a high Spearman’s rank correlation coefficient (*r*_s_ = 0.94). Spearman’s rank correlation is a non-parametrical alternative for Pearson’s correlation. It can be used to calculate the correlation between two variables that do not have normal distribution. What is more Spearman’s rank correlation is resistant for outlier results [31]. The correlation between ^210^Po and ^210^Pb is significant what confirms that source of these two radioisotopes in water is similar and no phosphogypsum impact is present. Many authors show that the value of ^210^Po/^210^Pb activity ratio higher than 1.0 must be due to three bio-geochemical processes which control the distribution of these radionuclides: rapid and selective scavenging of ^210^Pb by terrigenous particles; bioaccumulation of ^210^Po in the plankton and marine organisms which are recycled in the nutrient rich water; removal of both ^210^Po and ^210^Pb from the water column to the sediments so in future more reactive ^210^Po is subsequently released from the sediment into the water. The second and third processes are favored as being the more important [20].

**Table 1:** ^210^Po, ^210^Pb concentrations and ^210^Po/^210^Pb activity ratio in collected river water samples (given with expanded standard uncertainty calculated for 95 % confidence interval; *n* = 3)

Collection site	^210^Po concentration [mBq L^−1^]	^210^Pb concentration [mBq L^−1^]	^210^Po/^210^Pb activity ratio
1	0.8 ± 0.1	1.2 ± 0.1	0.67 ± 0.07
2	0.9 ± 0.1	1.5 ± 0.1	0.63 ± 0.09
3	0.9 ± 0.1	1.3 ± 0.1	0.69 ± 0.10
4	0.8 ± 0.1	1.4 ± 0.1	0.62 ± 0.07
5	2.0 ± 0.1	3.2 ± 0.1	0.62 ± 0.05
6	1.0 ± 0.1	1.6 ± 0.1	0.66 ± 0.07

### Uranium (^234^U, ^235^U and ^238^U) analysis

**Fig. 5:**
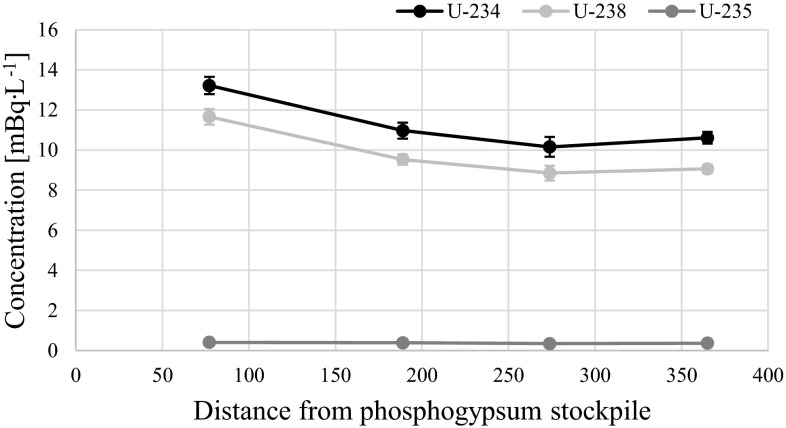
^234^U, ^238^U, ^235^U concentrations in river gradient

The activities of ^234^U, ^235^U and ^238^U radioisotopes in the Martwa Wisła river are presented in Table 2. ^234^U concentrations in analyzed water ranged from 10.2 ± 0.3 to 13.2 ± 0.4 mBq L^−1^, ^235^U concentrations lied between 0.35 ± 0.14 and 0.41 ± 0.10 mBq L^−1^, while ^238^U concentrations ranged from 8.8 ± 0.2 to 11.7 ± 0.3 mBq L^−1^ (Table 2). Uranium radioisotopes are mostly associated with solution due to the fact that in freshwater uranium is mainly present in soluble forms [32]. On the other hand, uranium sorption on clay minerals at pH < 5 and on Fe and Al hydroxides or silicas at pH > 5, depending on these compounds presence in water, can slightly decrease uranium concentrations [33]. Exactly opposite than with ^210^Po and ^210^Pb radionuclides, the highest concentration of diluted uranium radioisotopes were measured in the sample number 2 collected in the vicinity of the stockpile (Table 2; Figs. 1, 5). The measured concentration for this sample is 11.7 ± 0.3 mBq L^−1^ for ^238^U, 13.2 ± 0.4 mBq L^−1^ for ^234^U, 0.41 ± 0.10 mBq L^−1^ for ^235^U and 0.951 ± 0.001 µg L^−1^ for total uranium. Based on Q-Dixon test (*α* = 0.05) we can assume that uranium radioisotopes in this sample have different. The average ^238^U, ^234^U, ^235^U and total uranium concentrations for the rest of the samples were: 9.2 ± 0.3, 10.7 ± 0.5, 0.36 ± 0.02 mBq L^−1^ and 0.754 ± 0.028 µg L^−1^, respectively. There is a possibility that increased uranium concentration could be due to phosphogypsum leachates, especially after heavy rains. There are several studies and hypothesis that confirm that long-term leaching occurs when rainwater infiltrates through stack and that different types of water can elute uranium with varied efficiency. In general uranium is known to be soluble even in unperturbed river waters and is mostly present in <0.45 µm fraction of water [34, 35].

**Table 2:** ^234^U, ^238^U, ^235^U concentrations and ^234^U/^238^U, ^235^U/^238^U activity ratio in collected river water samples (given with expanded standard uncertainty calculated for 95 % confidence interval; *n* = 3)

Collection site	^234^U concentration (mBq L^−1^)	^238^U concentration (mBq L^−1^)	^235^U concentration (mBq L^−1^)	Total uranium (µg L^−1^)	^234^U/^238^U activity ratio	^235^U/^238^U activity ratio
1	11.1 ± 0.4	9.4 ± 0.4	0.37 ± 0.10	0.764 ± 0.001	1.18 ± 0.10	0.039 ± 0.011
2	13.2 ± 0.4	11.7 ± 0.3	0.41 ± 0.10	0.951 ± 0.001	1.13 ± 0.03	0.036 ± 0.008
3	11.0 ± 0.5	9.5 ± 0.4	0.39 ± 0.15	0.777 ± 0.001	1.15 ± 0.01	0.041 ± 0.015
4	10.2 ± 0.3	8.8 ± 0.2	0.35 ± 0.14	0.722 ± 0.001	1.15 ± 0.06	0.039 ± 0.015
5	10.6 ± 0.5	9.1 ± 0.4	0.37 ± 0.16	0.739 ± 0.001	1.17 ± 0.07	0.041 ± 0.018
6	10.8 ± 0.4	9.4 ± 0.3	0.39 ± 0.18	0.769 ± 0.001	1.15 ± 0.01	0.041 ± 0.018

**Fig. 6:**
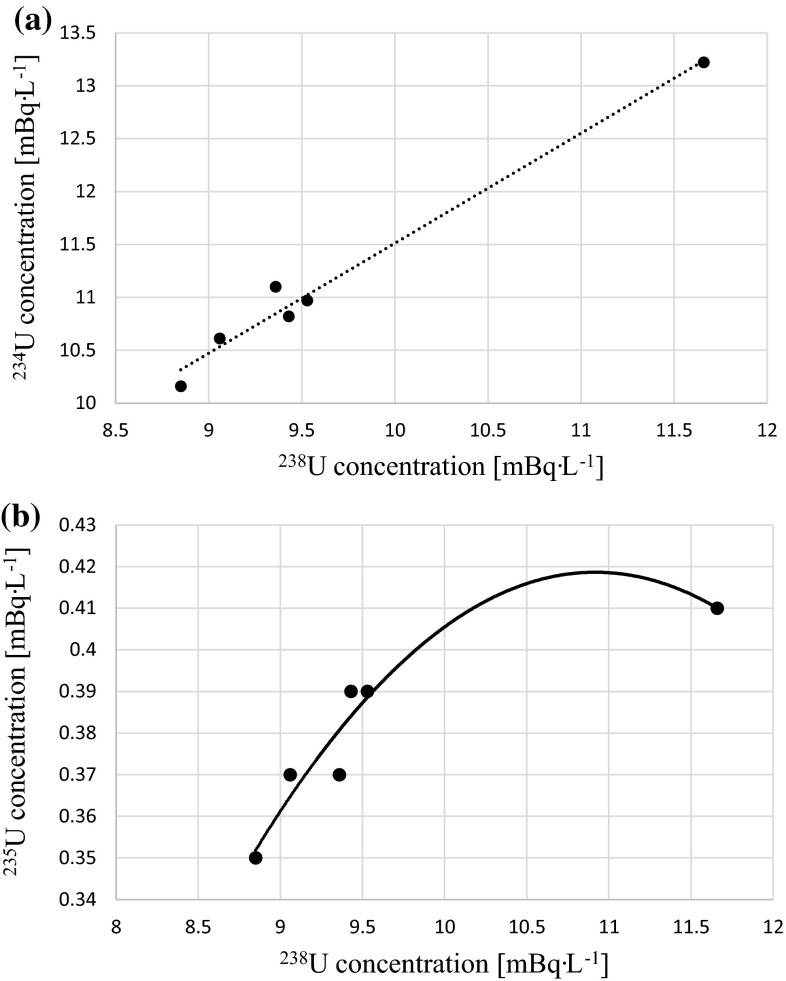
Correlation between: **a** ^234^U and ^238^U (*r*_s_ = 0.83) and **b** ^235^U and ^238^U (*r*_s_ = 0.97) concentrations in analyzed water samples

The other research conducted in Florida showed that within stockpiles uranium is mainly present as complexes with sulphate and phosphate, which are relatively mobile uncharged or negatively charged species. The most effective uranium elution from phosphogypsum occurs with saline water (e.g. seawater). Rainwater is known to elute less uranium as its conductivity is much lower compared with river water [36]. Lysandrou and Pashalidis found that stack solutions and leachates have different predominant uranium (VI) species. In case of acidic stack solutions (ph < 5) phosphate forms are dominant. On the other hand in leaching solutions (pH ≥ 5) dominant are fluoride complexes [36, 37]. However, under normal atmospheric conditions (pH > 7) carbonate uranium (VI) complexes are predominant [38]. According to these hypotheses and the fact that normal rain water has pH = 5.6 while pH for the Martwa Wisła river is 8.1 we can assume that uranium should exist in fluoride and carbonate forms. According to Papanicolaou et al. pH in range 4–8 does not affect phosphogypsum solubility [39]. The values of ^234^U/^238^U and ^235^U/^238^U activities ratios in the Martwa Wisła river lie between 1.13 ± 0.03 and 1.18 ± 0.01, and between 0.036 ± 0.008 and 0.041 ± 0.018, respectively (Table 2) and are typical for surface river water. Both ^234^U and ^235^U concentrations show high Spearman’s rank correlation coefficient with ^238^U (*r*_s_ = 0.83 for ^234^U and ^238^U, and *r*_s_ = 0.97 for ^235^U and ^238^U) (Fig. 6). These correlations are significant and suggest natural origin of these radioisotopes. Even though we can see that correlation factor for ^235^U and ^238^U concentrations is higher than for ^234^U and ^238^U. It is connected with fact that ^235^U concentrations in natural environment is low. This way alpha spectrometric measurement of this radioisotope is subject to greater uncertainty. On Fig. 6b we can clearly see that ^235^U and ^238^U relationship is not linear. Spearman’s rank correlation gives high correlation when the two variables being compared are monotonically related, even if their relationship is not linear. Probably if more samples were analyzed this relationship would be linear. ^234^U/^238^U disequilibrium is typical for river waters due to two mechanisms: *α*-particle recoil ejection of ^234^Th (a precursor of ^234^U) into solution and the preferential chemical solution of ^234^U due to the radiation damage of the crystal lattice caused by the decay of the parent ^238^U and subsequent decays results in ^234^U higher solubility in U^6+^ state [40–42]. Previous results obtained in 2008 for uranium (^234^U and ^238^U) activities measurements in water samples conducted in the area of phosphogypsum stack in Wiślinka suggest that in the Martwa Wisła river uranium concentrations were almost three times higher than today (from 25.34 ± 0.99 to 33.63 ± 1.61 mBq L^−1^ for ^238^U and from 25.38 ± 0.99 to 32.55 ± 1.65 mBq L^−1^ for ^234^U with ^234^U/^238^U activity ratio close to 1.00). This could be connected with two facts. First of all, after the phosphogypsum stockpile was closed, its recultivation started. The stockpile was covered with sewage sludge and common osiers (*Salix viminalis*) were planted close to the stack and the Martwa Wisła river. Stockpile covering has significant impact on decreasing phosphogypsum particles mobility [2, 43]. Common osier (*Salix viminalis*) is plant known for being hyperaccumulants of heavy metals [44]. Their plantations could probably have substantial effect on decreasing radionuclides and heavy metals concentrations in stockpile leachates. Secondly, there are many factors affecting uranium concentrations in river waters like mining or fertilizers use. What is more in 2008 general inflow of uranium was higher because long term rainfalls caused floods in Poland. We have to take into account that the Martwa Wisła is one of the last parts of the biggest Polish river Vistula. In this case the Martwa Wisła can collect radioisotopes from the whole Vistula’s basin. In pumping station and retention reservoir measured activities were significantly higher: 250 ± 7 for ^238^U, 248 ± 7 for ^234^U and 13,440 ± 68 for ^238^U, 13,140 ± 65 for ^234^U, respectively with ^234^U/^238^U activity ratios slightly less than 1.00 [6, 8]. These results suggested significant impact of the phosphogypsum waste heap in Wiślinka. The uranium ^234^U, ^238^U and ^235^U radioisotopes concentrations received in present survey are similar to those reported by other researchers. One of the exceptions are water samples collected by Jurado Vargas et al. from the Ortigas river in the granitic southwest region of Spain. The Ortigas river passes through the mines and this region is known for high uranium activities. The ^234^U, ^235^U and ^238^U concentrations reached maximal concentrations of 1200 ± 60, 51 ± 4 and 2000 ± 100 mBq L^−1^, respectively [40]. The average values for the Ortigas river were almost four times higher than for the Martwa Wisła river with ^235^U/^238^U activity ratios similar to those received by us. The authors presented higher ^234^U/^238^U disequilibrium with maximal value of 1.77 ± 0.09. They explain it by the fact that the Ortigas river water had static and quasi-dynamic conditions what intensified the water contact time with soil thereby increasing the importance of exchange of uranium contained in the sediments. General trend of increased ^234^U/^238^U activity ratio with concentration was observed in static water condition [40]. This fact is not confirmed by our research. The Martwa Wisła is considered to be static river. Even though the values of ^234^U/^238^U activity ratios received by us are much lower than in the static the Ortigas river. This fact might be connected with uranium containing fertilizers leachates from agricultural lands located close to the Martwa Wisła river along all its bank [40]. Vidic et al. measured ^234^U and ^238^U concentrations and their activity ratios in spring waters from Bosnia and Herzegovina where depleted uranium ammunition was employed in 1995. No significant impact of DU was determined and the uranium activities (1.85 ± 0.25 to 13.9 ± 0.71 mBq L^−1^ for ^238^U and from 3.20 ± 0.23 to 15.3 ± 0.76 mBq L^−1^ for ^234^U with mean value of 1.37 for ^234^U/^238^U activity ratio) were very similar to our results (Table 3). The highest uranium concentrations noticed in Bosnia and Herzegovina may be connected with geological composition of the investigated area. The substrate of the river is represented by sandstones, argillites, clayey marls and sandy limestones [34]. Other authors show that leaching of uranium from these materials is more efficient [45, 46] What is more the narrower range for both ^234^U and ^238^U in the Martwa Wisła river than in Bosnia and Herzegovina is probably connected with the fact that our sample collection sites were located close to each other what was necessary in order to examine the impact of the phosphogypsum stockpile. In Bosnia and Herzegovina samples were collected from many locations along the entire river course. This way each sample collection site could be exposed to different uranium sources what can explain the wide range of these results. Comparing the Martwa Wisła results with ^238^U concentrations in major Polish rivers we find that for the Odra river obtained results were from 5.45 ± 0.14 to 14.69 ± 0.37 mBq L^−1^, while for its tributaries the uranium activities were from 2.59 ± 0.04 to 13.06 ± 0.44 mBq L^−1^ [47]. ^238^U concentrations in unfiltered water of the Vistula river in autumn were from 6.34 ± 0.43 to 15.12 ± 0.23 mBq L^−1^. In the Vistula tributaries the uranium concentrations ranged from 5.94 ± 0.19 to 11.95 ± 0.21 mBq L^−1^ (Table 3). The values of ^234^U/^238^U activity ratios lied between 1.10 ± 0.07 and 1.74 ± 0.20 for the Vistula and between 1.02 ± 0.08 and 1.59 ± 0.08 for its tributaries [48]. The obtained differences between the Martwa Wisła river and the Vistula and the Odra rivers are the result of removing of mine waters, the use of phosphate fertilizers in agriculture, increased surface and underground runoff, the discharge of saline mine water and increased soil erosion. These differences are also a result of the processes of weathering and erosion of the Sudetes rocks, which contain elevated natural concentrations of uranium. What is more anthropogenic uranium in this area is the result of coal mining in the Lower Basin [46]. It is worth noticing that differences in the ranges of ^234^U and ^238^U concentrations compared to the Martwa Wisła river can be also explained by the fact that samples from the Odra and the Vistula rivers, and their tributaries were collected from multiple locations along their entire courses.

**Table 3:** ^234^U and ^238^U concentrations comparison in different rivers

River	^234^U concentration (mBq L^−1^)		^238^U concentration (mBq L^−1^)		Reference
	Min	Max	Min	Max	
Vistula river	8.15 ± 0.49	17.8 ± 0.25	6.34 ± 0.43	15.12 ± 0.23	Skwarzec et al. [46]
Vistula tributaries	6.18 ± 0.36	15.56 ± 0.25	5.94 ± 0.19	11.95 ± 0.21	
Odra river	9.28 ± 0.19	27.08 ± 0.50	5.45 ± 0.14	14.69 ± 0.37	Skwarzec et al. [45]
Odra tributaries	3.52 ± 0.05	19.92 ± 0.95	2.59 ± 0.04	13.06 ± 0.44	
Bosnia and Herzegovina	3.20 ± 0.23	15.30 ± 0.76	1.85 ± 0.25	13.90 ± 0.71	Vidic et al. [33]
Martwa Wisła	10.2 ± 0.3	13.2 ± 0.4	8.8 ± 0.2	11.7 ± 0.3	Present study

## Conclusions

In this survey we tried to establish the real impact of the possible leachates to the Martwa Wisła river from phosphogypsum stockpile in Wiślinka. Our results for diluted fraction of ^210^Po, ^210^Pb, ^234^U, ^235^U and ^238^U radioisotopes suggest that this impact is rather not significant. When compared to the results obtained in previous surveys conducted in this area, the obtained results are lower which leads to the conclusion that present process of stockpile recultivation is probably partially successful. We cannot be sure if increased values of more soluble uranium radioisotopes in close vicinity to stockpile is not connected with possible leachates. In this case we have to admit that stockpile is probably not completely secured from soluble elements that are leached into the Martwa Wisła river. Nevertheless, these leachates are directly diluted in the Martwa Wisła river water.
